# Supplementary material for: Physiological effects of alfaxalone anesthesia on rhesus monkeys during intravenous glucose tolerance testing
Source: PLoS One. 2024 Jul 26;19(7):e0308007. doi: 10.1371/journal.pone.0308007 (PMC11280158; doi:10.1371/journal.pone.0308007)
Supplement: S1 Table — (DOCX) [file pone.0308007.s001.docx]

**S1 Table: Individual subject demographics**

| Animal | Sex | Age (yrs) | Weight (kgs) | BCS^*^ |
| --- | --- | --- | --- | --- |
| MK1 | F | 5 | 7.85 | 4/5 |
| MK2 | F | 5 | 6.98 | 3.5/5 |
| MK3 | F | 6 | 6.38 | 3/5 |
| MK4 | F | 6 | 9.25 | 3.5/5 |
| MK5 | F | 19 | 8.76 | 3/5 |
| MK6 | M | 6 | 9.24 | 3/5 |
| MK7 | M | 7 | 9.67 | 3/5 |
| MK8 | M | 12 | 8.47 | 2/5 |
| MK9 | M | 16 | 12.07 | 3.5/5 |
| MK10 | M | 17 | 9.96 | 3/5 |

*Body condition score
