# Supplementary material for: Physiological effects of alfaxalone anesthesia on rhesus monkeys during intravenous glucose tolerance testing
Source: PLoS One. 2024 Jul 26;19(7):e0308007. doi: 10.1371/journal.pone.0308007 (PMC11280158; doi:10.1371/journal.pone.0308007)
Supplement: Attachment — Submitted filename: Review 5-3-24.pdf [file pone.0308007.s002.pdf]

This manuscript assessed the effects of alfaxalone anesthesia on rhesus macaques during IV glucose tolerance testing. The study compared IVGTT results, need for supplemental doses, sedation depth, BP, HR, SPO2, induction time, recovery time between 4 different combination analgesic regimens. All included the use of glycopyrrolate (0.015 mg/kg) and either Telazol (5mg/kg), alfaxalone (7.5 mg/kg), alfaxalone (12 mg/kg), alfaxalone (15 mg/kg). It was assessed the combination of alfaxalone (12 mg/kg) and glycopyrrolate (0.015 mg/kg) was the ideal dose for IVGTT.

Minor edits/suggestions:

- Since Glycopyrrolate was used for each anesthetic study group it should be stated that it is used; this study should be an assessment of four combination anesthetic regimens throughout this manuscript (abstract, introduction (Line 81-84), and discussion (Line 249). Considering glycopyrrolate can cause false increase of HR, increase of temp, hyposalivation, it can greatly effect the parameters of the results, and the anesthetic groups (Telazol, Alf7.5, Alf12, and Alf15) should be assessed as a combination anesthetic regimen throughout the study.
- Line 19, add word for clarity: “to three different doses of alfaxalone”
- Add citations for lines 40, 50, and 71.
- Low animal number in study; due to this study evaluating cardiovascular parameters and an sedative/anesthetic it would be helpful to provide Table 1 with body condition scores of the group and list out for each monkey its age, sex, weight, and body condition score.
- Line 107-108 list route for sedatives
- Line 265-267: This is a promising find for alfaxalone as it lowers the anesthesia risk potential and may reduce the need for extra sedation time to treat effects of salivation
    - Cannot really conclude on salivation for alfaxalone-considering all animals were given Glycopyrrolate which is an antisialogogue, unless an animal group only received alfaxalone without glycopyrrolate.
- Did Alf12 and Alf15 have shorter recovery times because of the actual anesthetic regimen used or was it more likely due to not receiving supplemental doses? Should discuss this in the discussion.
- Probably should include comparing some of your results with this study in the discussion:
    - Wada S, Koyama H, Yamashita K. Sedative and physiological effects of alfaxalone intramuscular administration in cynomolgus monkeys (Macaca fascicularis). J Vet Med Sci. 2020 Jul 31;82(7):1021-1029. doi: 10.1292/jvms.20-0043. Epub 2020 May 26. PMID: 32461537; PMCID: PMC7399308.
